# Fractionated Antioxidant and Anti-inflammatory Kernel Oil from *Torreya fargesii*

**DOI:** 10.3390/molecules24183402

**Published:** 2019-09-19

**Authors:** Xianrong Zhou, Jin Shang, Mingyi Qin, Jianhua Wang, Bo Jiang, Hui Yang, Yan Zhang

**Affiliations:** School of Advanced Agriculture and Bioengineering, Yangtze Normal University, Fuling 408100, Chongqing, China; shangjin2017@outlook.com (J.S.); mingyiqin2018@outlook.com (M.Q.); jianhuawang2017@outlook.com (J.W.); BoJ2015@163.com (B.J.); hyytnu@outlook.com (H.Y.); Yanzhang123@163.com (Y.Z.)

**Keywords:** *Torreya*, kerneloil, sciadonic acid, antioxidant effect, anti-inflammation

## Abstract

**Abstract:**

Polymethylene-interrupted polyunsaturated fatty acids (PMI-PUFAs) are emerging functional lipids with proven antioxidant and anti-inflammatory effects. In this study, a typical PMI-PUFA, sciadonic acid (C20:3, 5c 11c 14c), was enriched in the kernel oil of *Torreya fargesii* (*T. fargesii*) by fractionation. Fractionated kernel oil of *T. fargesii* (containing 25% sciadonic acid) showed equal stability and similar radical scavenging ability compared with the non-fractionated oil. In anti-inflammatory tests, fractionated kernel oil was shown to inhibit the activity of phosphodiesterase (PDE-5, efficiency 80% at 133.7 μg/mL) and lipoxygenase-5 (LOX-5, efficiency 65% at 66.7 μg/mL) more effectively than the non-fractionated oil. This shows that increasing the amount of sciadonic acid can enhance the anti-inflammatory effect of the kernel oil. This research also indicates that fractionation is a feasible way to obtain sciadonic acid–rich functional oil with potential pharmacological effects.

**Practical Applications:**

This study proved that fractionation can effectively enrich the content of PMI in *Torreya* kernel oil and improve its anti-inflammatory biological activity. It is a less expensive and possible mass-produced method to obtain PMI-PUFA–enriched *Torreya* oil. It is helpful in reducing the addition of *Torreya *oil in products and providing better bioactivity. Further studies are expected on the anti-inflammatory effects of enriched PMI-PUFA oil in vivo and the possibility of enriching other PMI-PUFA oils by fractionation.

## 1. Introduction

**Figure 1:**
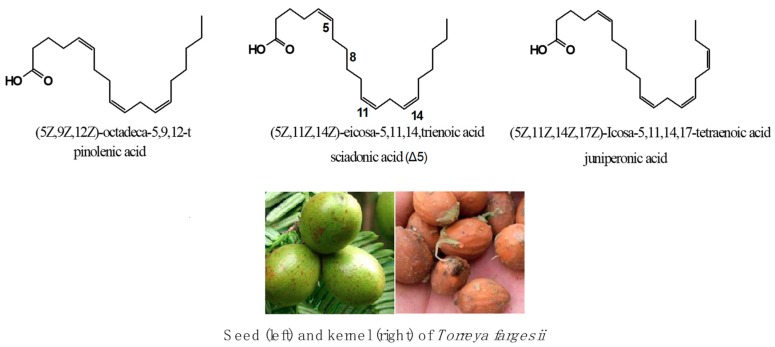
The typical polymethylene-interrupted polyunsaturated fatty acids (PMI-PUFA) and seed of *Torreyafargesii.*

Polymethylene-interrupted polyunsaturated fatty acids (PMI-PUFAs) refer to a series of unusual fatty acids that are different from normal Δ_3_/Δ_6_/Δ_9_ PUFAs. PMI-PUFAs have a unique structure of two or more double bonds (in *cis*-configuration) separated by at least one polymethylene group [1]. Natural PMI-PUFAs include pinolenic acid (C18:3, 5c 9c 12c), sciadonic acid (C20:3, 5c 11c 14c), Δ_7_-eicosatrienoic acid (C20:3, 7c 11c 14c), and juniperonic acid (C20:4, 5c 11c 14c 17c) (Figure 1). In commercial applications, fat with pinolenic acid was used as an ingredient in diet food [2] and structured lipids [3]. At an academic level, Pedrono found that sciadonic acid has anti-hypertriglyceridemic effects due to its ability to inhibit on Δ_9_ desaturase [4]. Similar studies showed that sciadonic acid can reduce rats’ serum and liver triacylglycerol levels [5]. Huang and Chen [6,7] found that sciadonic acid has a strong anti-inflammatory ability; the mechanism may be inhibition of COX-2activity or reduction of pro-inflammatory mediators. It is still of value to determine further properties of the anti-inflammatory activity of sciadonic acid.

PMI-PUFAs are found in the kernels of gymnosperms [8,9] such as Podocarpaceae, Pinaceae, and some Taxaceae plants. *Torreya* is a genus of Taxaceae. *Torreya grandis* cv. *merrillii* (*T. grandis*) is mainly distributed in Southeast China.Its kernel oil contains about 10–13% sciadonic acid [10,11,12]. Recently, several studies reported that kernel oil of *T. grandis* has strong antioxidant activity in radical scavenging tests [13,14,15]. *Torreya fargesii* (*T. fargesii*) is an endemic species that grows in the mountainous areas of Central and Southwest China. The botanical classification and ecological habit of *T. fargesii* differ from those of *T. grandis* [16]. Our previous work [17,18] indicated that flavonoids or polyphenols extracted from the arils of *T. fargesii* have antioxidant activity. However, studies of kernel oil from *T. fargesii* are scarce compared to studies of *T. grandis*.

The content of PMI-PUFA contributes to the pharmacological activity and nutritional value of *Torreya* oil and its final commercial products [6,7]. We noted that the kernel oil of *Torreya* species contains about 10% saturated fatty acid and 20%–30% monounsaturated oleic acid [11,12], which is suitable for concentrating their unsaturated fatty acids by fractionation. In this work, we investigated the use of conventional methods of fractionation to produce *Torreya* oil with high anti-inflammatory activity. The stability and antioxidant efficiency of fractionated (FT) and nonfractionated (UT)kernel oil of *T. fargesii* was tested by heating acceleration, DPPH, and Oxygen Radical Absorbance Capacity (ORAC) tests. We also studied the activity of the kernel oil on inflammatory-related phosphodiesterase (PDE-5) and lipoxygenase-5 (LOX-5) for the first time.

## 2. Result and Discussion

### 2.1. Kernel Oil of T. fargesii and its Properties

The total lipid content in the *T. fargesii* kernels was 50.3 ± 2.6%. The oil content in the *T. grandis* kernels ranged from 45% to 55% depending on the growth region [10,11,12,14]. The oil content of the kernels was comparable to that of rapeseed and sunflower seed (about 35–50%).

Table 1 lists the range of physiochemical parameters of *T. grandis* from the literature. UT oil has higher content of stearic acid, linoleic acid, and saturated fatty acids than kernel oil from *T. grandis*. Therefore, it is possible to enrich sciadonic acid by separating the saturated fatty acids from UT oil. There was no obvious difference between UT oil and other kernel oils of *T. grandis* (*p* < 0.05) for other physicochemical properties and fatty acid composition. Kernel oil from *Torreya* species contained at least 1500 ppm (1500 mg/kg) of tocopherols, which is higher than common soybean oil/rapeseed oil (usually 100–500 ppm).

### 2.2. Dewaxing and Fractionation

UT oil contains about 2% wax (Table 2). After dewaxing (WP), the wax content was reduced to 0.45%, with a 95–98% yield of dewaxing oil. The sciadonic acid content, melting range, and SFC content did not change during dewaxing (*p* < 0.05; Table 2). The DSC curve (Supplementary Figure S1) showed two main exothermic processes at about 15–20 °C and 0–5 °C, indicating that two groups of solid triglycerides can co-crystallize from oil [19]. Thus, the fractionation included a one-step dewaxing and a two-stage cooling process. The one-stageFP1 (from 30 °C to 5 °C, cooling rate 0.5 °C/h) and two-stageFP3 (rate 1 °C/h, 30–15 °C; 0.5 °C/h, 15–5 °C) showed similar results in yield (75–76%) and sciadonic acid content (25%) in the liquid fraction; one-stageFP2 (1 °C/h) decreased the sciadonic acid content (15%) in the liquid fraction. The change of stirring rate in FP4 decreased the sciadonic acid content. The liquid fraction ofFP3also showed a lower melting range than that of FP1; the lower melting point can keep the oil transparent in cold weather and is helpful in commercial applications. Based on comprehensive consideration, the two-stageFP3 process saves one-third of the cooling time (as well the energy consumption) over the one-stage cooling process. Therefore, FP3 is a suitable procedure in this study.

As a result, the sciadonic acid content increased from 11% to 25%; the yield of low-melting-point fraction (FT oil) reached 75%. Compared with UT oil (Table 1), FT oil had higher UFA, PUFA, and MUFA content, while the SFA content decreased from 15% (UT oil) to about 6% (FT oil). For other physicochemical properties, the increased unsaturated number (UFA content) resulted in a larger IV value in FT oil than that in UT oil. There was no significant difference in AV, PV, and saponification value before and after fractionation. Fractionation did not change the content of unsaponifiable matter, which often contains phytochemicals.

By comparison, stearidonic acid, a functional lipid, was concentrated to 29% in *Echium* seed oil by fractionation with urea encapsulation [20]; oil with high sciadonic acid content (60%) was isolated by combining enzymatic esterification and urea encapsulation from kernel oil of *T. grandis* [3]. The use of urea and enzymes increases production costs, and urea may cause harmful residues.

### 2.3. Stability of Fractionated Oil

**Figure 2:**
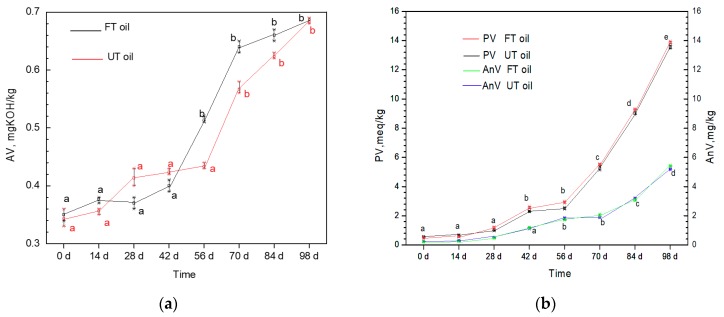
AV (**a**), PV and AnV (**b**) of UT oil and FT oil in the long-term storage test.

As shown in Figure 2, the AV of the two oil samples changed slowly in 42 days (*p* < 0.05) and the difference between FT oil and UT oil was not significant (*p* < 0.05), indicating that the increased sciadonic acid content did not affect the AV.

After a 3 month (98 d) test, the AV of FT oil and UT oil was about 0.7 mg KOH/kg. The moisture absorbed by oil samples from the air is a possible reason for increased AV. PV and AnV did not change significantly within 28 d (*p* < 0.05), and then increased rapidly after 56 d of storage. There was no difference in PV and AnV between FT oil and UT oil in 98 d (*p* < 0.05). The storage test under natural conditions can support potential commercial applications, such as best flavor period after opening, package details, and addition of antioxidants.

**Figure 3:**
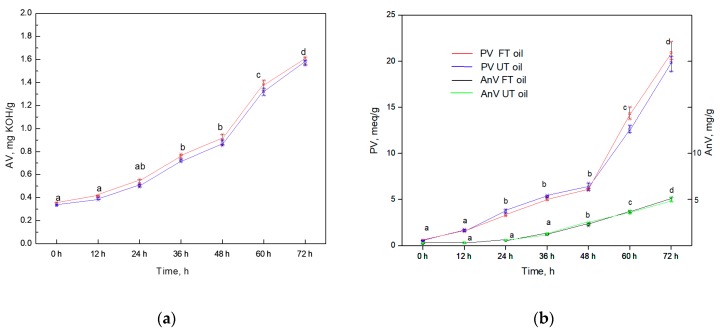
(**a**), PV and AnV (**b**) of UT oil and FT oil in a Schaal accelerated test. * The same lowercase letters means the no significant difference between values of different days in the same curve (*p* < 0.05). ** No significant difference between the curves of AV, PV and AnV for FT oil and UT oil (*p* < 0.05). *** Data are presented as mean ± SD, *p* < 0.05.

The Schaal test can reflect the oxidant sensitivity of PUFA under heating conditions. In 24 h, there was no significant difference in the change of Av and PV; no difference was found between FT oil and UT oil (*p* < 0.05; Figure 3). PV continually increased from about 1.0 mEq/g to 20.0 mEq/g over 72 h of heating. There was no difference in PV between FT and UT oil during the testing period (*p* < 0.05). The long-term and Schaal tests indicated that the stability of FT oil does not decrease as its PUFA content increases.This is partly attributed to the increased tocopherols content in FT oil (from 1830 to 2020 mg/kg).

### 2.4. Radical Scavenging Test 

#### 2.4.1. DPPH Scavenging Efficiency

The chemical mechanism of the free radical scavenging test usually includes single electron transfer (SET, such as DPPH) and hydrogen atom transfer (HAT, typical of ORAC) [21]. Results of the DPPH assay showed that the radical scavenging ability of FT oil was similar to that of UT oil (*p* < 0.05) at all concentrations. The scavenging efficiency was about 65% when the concentration of FT oil was higher than 8 mg/mL. As shown in Table 3, FT oil showed similar scavenging activity (502 ± 18 μmol TE/100 g) to UT oil (485 ± 15 μmol TE/100 g), which is consistent with their removal efficiency. The DPPH radical-scavenging activity and polyphenols content were highly correlated, and the polyphenols content was similar in FT and UT oil. The increasing content of tocopherols (61.6 μg TE/mL, about 24 μmol TE/100 g [22]) cannot influence the DPPH activity between FT oil and UT oil.BothUT and FT oil showed equal DPPH scavenging ability to the reported *Torreya* oils (between 422 and 509 μmol TE/100 g) [11,12,14]. The IC_50_ of FT and UT oil was about 6.0 μg/mL, lower than that of the chemically synthesized BHT (about 1.75 μg/mL) but stronger than the EGCG (90 μg/ML [23]). This indicates that the DPPH scavenging activity of FT oil was limited.

#### 2.4.2. ORAC Capacity 

As shown in Table 3, the methanol extract of FT oil had a higher ORAC value (468 μmol TE/100 g) than the UT oil (445 μmol TE/100 g) (*p* < 0.05), but their values are far below water-soluble epigallocatechin gallate(EGCG, about 8000 μmol TE/100 g).The reported ORAC values of *T. grandis* kernel oil varied from 260 to 435 μmol TE/100 g [12,14].The tocopherols, phytosterols, and polyphenols were highly indicative of the strong ORAC value of plant oil. In general, tocopherols usually accumulate in the liquid phase (FT oil) in fractionation. The ORAC of tocopherols varies from 1200 to 2000 μmol/g [24], so the increased tocopherols content (about 0.02 g increment in FT oil, 24–40 μmol TE/100g FT oil) contributed to the stronger ORAC of FT oil. Meanwhile, the stability of FT oil did not change as its PUFA content increased, due to increased tocopherols.

### 2.5. Anti-inflammatory Effect

#### 2.5.1. PDE Inhibition

**Figure 4:**
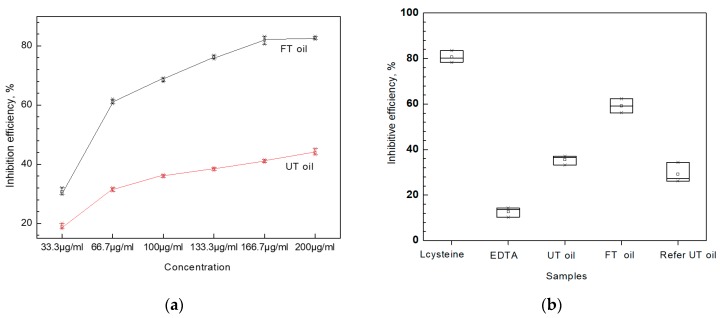
The inhibition efficiency of PDE-5 by UT oil and FT oil (**a**) and the comparison test with EDTA and L-cysteine (**b**), (data are presented as mean ± SD, *p* < 0.05).

The inhibition efficiency toward PDE-5 increased to over 80% with increased FT oil concentration to 133.7 μg/mL;a higher concentration of FT oil only increased efficiency to about 85% (Figure 4). FT oil with 25% sciadonic acid had stronger inhibition capacity than UT oil with 11% sciadonic acid UT oil (133.7 μg/mL) showed only about 40% inhibition efficiency, suggesting that the enrichment of sciadonic acid effectively enhances PDE-5 inhibition ability. The possible inhibition activity of tocopherols on PDE-5 can be excluded by the reference test, by which UT oil with 2000 mg/kg tocopherols showed only about 35% inhibition efficiency. We used EDTA and L-cysteine as positive and negative references, respectively, to evaluate PDE-5 inhibition activity. The efficiency between EDTA (about 80%) and L-cysteine (about 8%) was regarded as effectively anti-inflammatory. The inhibition efficiency of FT oil (133.7 μg/mL) was about 78%, indicating its potential anti-inflammatory value. As reference, the aqueous extract of nutmeg showed over 90% inhibition efficiency on PDE-5 at 500 μg/mL [25]; in a cell model test, East Indian sandalwood oil showed about 70% inhibition efficiency on PDEs ata concentration of 0.0001–0.0002% [26].

#### 2.5.2. LOX Inhibition

The inhibition of LOX-5 (Table 3) revealed that FT oil (inhibition efficiency 65.2%, with 25% sciadonic acid) was twice as effective as UT oil (32.1%, with 11% sciadonic acid). The 10% increased tocopherols content in FT oil cannot double the LOX-5 inhibition activity, indicating that the inhibition of LOX-5is related to the concentration of sciadonic acid in the kernel oil.The increased FT oil concentration in the test system could not increase the inhibition efficiency clearly, which was similar to the PDE-5 inhibition test. The inhibition efficiency of FT oil was about two-thirds of NGDA (stronger inhibitor of LOX enzymes), showing its potential value in anti-inflammatory applications. For comparison, the oil from *Psidiumguajava* fruits showed an IC_50_ of about 196 μg/mL on LOX-5 [27], and the essential oil from thyme species only showed 12% efficiency at 100 μg/mL [28].

#### 2.5.3. Inhibition of Mouse Ear Edema

**Figure 5:**
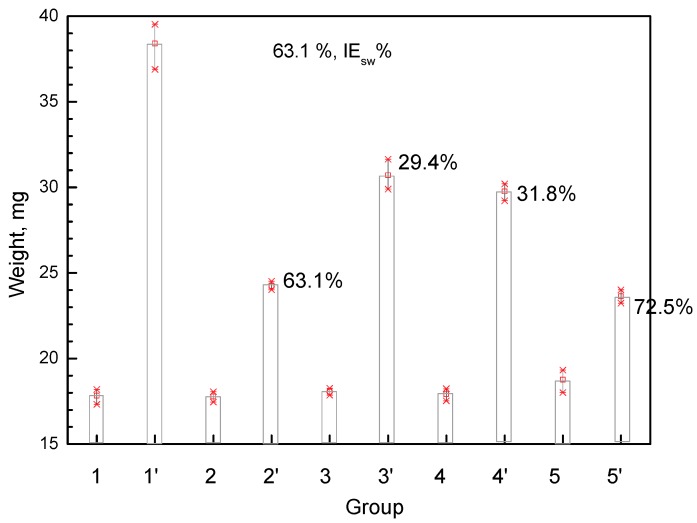
Effect of FT oil and UT oil on xylene induced ear inflammation in mice. (n = 15, Data are presented as mean ± SD, *p* < 0.05;1,2,3,4,5 right ear, 1′,2′,3′,4′,5′ left ear;.1,1′ control, 2,2′ with FT oil, 3,3′ with UT oil, 4,4′ with reference (UT oil with 2000 mg/kg tocopherols), 5,5′ with aspirin).

The inhibition of topical edema on mouse ears induced by xylene is shown in Figure 5. The right ears of mice developed discernible edema after xylene contamination. Treatment with FT oil significantly decreased the weight of edematous ears by 63.1%, which is higher than with UT oil (29.4%) but lower than the most effective aspirin (72.5%). UT oil with the same amount of tocopherols as FT oil (2000 mg/kg) only showed 31.8% inhibition. This indicated that the increased anti-swelling capacity is independent of the tocopherols content in FT oil. As a comparison, the volatile oil of *Houttuyniacordata* Thunb. was reported to have 67.6% inhibition efficiency on xylene-induced ear edema in mice [29], while research on pumpkin seed oil showed about 70% inhibition of edema induced by 0.02 mL xylene on each ear [30]. This indicates the practical anti-inflammatory effect of FT oil in commercial applications.

## 3. Conclusions

Fractionation can effectively increase the sciadonic acid content in kernel oil of *Torreya fargesii* from 11% to 25%. Fractionated kernel oil of *Torreya fargesii* has similar stability, physicochemical properties, and antioxidant capacity as non-fractionated kernel oil. In anti-inflammatory assays, fractionated kernel oil showed inhibition of PDE-5/LOX-5 enzymes and mouse ear edema better than non-fractionated oils.This study provides an economical method for enriching sciadonic acid from the kernel oil of *Torreya fargesii* and demonstrates that oil rich in sciadonic acid has enhanced anti-inflammatory capacity.In this respect, further research on the mechanism of anti-inflammatory and other pharmacological effects of sciadonic acid and kernel oil may be of interest.

## 4. Materials and Methods

### 4.1. Chemicals

The mixed standard of fatty acid methyl esters (18916-1AMP), 2,2-diphenyl-1-picrylhydrazyl (DPPH), 2,2′-azobis(2-methylpropionamidine) dihydrochloride (AAPH), (±)-6-hydroxy-2,5,7,8-tetra-methylchromane-2-carboxylic acid (Trolox), squalene (442785), α-cholestane, disodium (4-nitro-phenyl) phosphate (4-NPP), 5-lipoxygenase (L6632), and phosphodiesterase (P4631) were purchased from Sigma-Aldrich (St. Louis, MO, USA). Pure water was produced by a Milli-Q Academic A10 system (Millipore, Burlington, MA, USA). Other reagents (in analytical grade) were purchased from Sinopharm (Shanghai, China).

### 4.2. Animals

Kunming mice (17–22 g) were purchased from HFK Bioscience Technology (Beijing, China). They were placed in plastic boxes at about 25 °C and fed for 15 days in a laboratory environment before the test. The animal experiments were approved by the Laboratory Animal Ethics Committee of Chongqing University (permit number: 2019121).

### 4.3. Preparation of Kernel Oil of T. fargesii

The seeds of *T. fargesii* were gathered at Daba Mountain (Chongqing Municipality, China) in October 2018. The kernels were obtained after peeling off arils and subsequently dehulling shells. After desiccating at 30 °C in vacuum for 48 h (moisture 3%–6%), the prepared kernels were crushed to about 5–10 mm size (using a sieve to control the particle size). Then the crushed kernels were pressed in a screw-type oil expeller (YZY-X 12 P, Kangyuan Sinomech., Beijing, China). The prepared crude oil was centrifuged at 8000 rpm for 15 min. The clear upper oil phase was collected and stored in a refrigerator at 4 °C. 

### 4.4. Physicochemical Properties of Kernel Oil 

The oil content of the kernels and meal was determined by the American Oil Chemists’ Society (AOCS) method Am 2-93. The physicochemical properties of the oil sample, including wax content (Ch 8-02), moisture and volatile matter (Ba 2a-38), acid value (AV, Cd 3d 63), peroxide value (PV, Cd 8b-90), unsaponifiable matter(Ca 6a-40), iodine value (IV, Tg1a-64), saponification value (SV, Cd 3c-91), fatty acid composition (Ce 1a-13), tocopherols (Ce 8-89), and phytosterols content (Ce 12-16), were measured using the official standardized AOCS methods [31].Viscosity was tested on a DV2T viscometer(Brookfield, Toronto, Canada).

#### 4.4.1. Total Polyphenols Content

Using a modified method [11], 1 g oil (diluted with 7 mL hexane) was loaded on a 500 mg Sepax Generik Diol column (Sepax Technologies, Newark, DE, USA). The column was successively washed with 6 mL of *n*-hexane, 2 mL of ethyl acetate, and 10 mL of methanol. These eluants were combined and 3 mL was orderly mixed with 0.5 mL of Folin–Ciocalteu reagent and1 mL of sodium carbonate solution (15%), and then metered to 10 mL with water. After 2 h incubation in a dark place, the prepared solution was measured at 760 nm on a UV 2700 spectrometer (Shimadzu, Kyoto, Japan) using gallic acid as a reference. The result was expressed as gallic acid equivalent (GAE) per kilogram of oil.

#### 4.4.2. Squalene

An oil sample (0.5 g) was saponified with 50 mL KOH/ethanol solution (2 mol/L) at 85 °C for 1 h and then extracted by 25 mL hexane three times. The hexane extracts were combined and washed with ethanol and dried with anhydrous Na_2_SO_4_. The as-prepared sample was tested on a Shimadzu GC-9A chromatograph equipped with a Stabilwax-DA capillary column using pure squalene as the internal standard; the other details were similar to a previous study [32].

### 4.5. Dewaxing and Fractionation 

About 500 g of oil sample was cooled using the preset cooling program in a cryotrap (DC0506 Shanghai Jingying Co.,Ltd., Shanghai, China). In the dewaxing process, the UT oil was cooled from 40 °C to 25 °C at 2 °C/h steps without stirring. After the 2 h settlement at 25 °C, the precipitated wax was filtrated from the oil under cooling conditions. The cooling program referred to the DSC analysis of UT oil and a published document [33]. The fractionation was set as a1-stage or 2-stage crystallization with different cooling and stirring rates. After each cooling process, the oil mixture was kept at the final temperature for 4h, then the liquid fraction (FT oil) and solid fraction were separated by centrifugation (10,000 rpm, 5 min). In the 2-stage crystallization, the liquid fraction from the first crystallization was used for a further cooling process. The solid fat content (SFC) of fractions was measured by NMR (AOCS Cd 16b-93) on an MQC micro-NMR spectrometer (Oxford, England, UK). The wax and sciadonic acid content, and melting range (AOCS Cc 1-25) were also measured to evaluate the efficiency of fractionation.

### 4.6. Stability of Fractionated Oil

In the 98-day long-term storage test, a 200 mL oil sample was metered in an opened plastic bottle, which was placed at room temperature with natural light irradiation. Then 5 mL of oil was pipetted every 14 days to determine its acid value (AV), peroxide value (PV), and anisidine value (AnV). In the Schaal test, a 250 mL oil sample in a glass flask was heated in an oven at 63 (±1) °C. PV, AV, and AnV were measured every 8 h, and the overall heating time was 72 h.

### 4.7. DPPH and ORAC assays

#### 4.7.1. DPPH

DPPH radical scavenging ability of the oil samples was assessed according to the reported method [34]. Typically, different amounts of test oil solution (100 mg/mL in ethyl acetate) and 2 mL of 0.1 mmol/L DPPH reagent were diluted with ethyl acetate to volume. After incubation in a dark place for 2 h at room temperature, the absorbance of the reactant system was measured at 515nm on a Shimadzu UV 2700 spectrophotometer with a 1 cm quartz cuvette. Trolox solution (in ethyl acetate) was used to prepare the work curve. 2,6-di-*ter*t-Butyl-4-methyl phenol(BHT) and ethanol were the positive control and reagent blank, respectively. The scavenging efficiency (E%) of DPPH radicals was calculated using the following equation:

(1) $$E\%=(1-\frac{A_{1}}{A_{0}})\times 100\%$$

where *A*_1_ and *A*_0_ are the absorbance of the reactant solution and the reagent blank, respectively. Antioxidant activity was also expressed as antioxidant equivalent to Trolox (an analogue of vitamin E, μmol TE/100 g of oil).

#### 4.7.2. ORAC

The method was modified from published research [35]. The oil sample (50 mL) was first extracted with an equal volume of 80% methanol for15 min in an ultrasonic bath, then the methanol layer was collected by centrifugation (8000 rpm, 10 min). For a typical measurement, FL reagent (150 μL, 0.08 μmol/L) in phosphate-buffered saline (PBS) buffer (pH = 7.4) and 20 μL of methanol extract of oil sample was mixed on a 96-well plate. The mixture was shaken for 3 min and incubated at 37 °C in the dark for 15 min, and then the AAPH solution (25 μL, 150 mM) was added to initiate the reaction. The decay of fluorescent emission was measured at 37 °C every 3 min at 525 nm (excitation at 485 nm) using a Multiskan MK3 reader (Thermo, Waltham, MA, USA). ORAC of test samples was determined by using Trolox as a standard; the integral areas of fluorescence peaks from samples and Trolox were calculated by the software. The result was expressed as μmol Trolox equivalent/g (μmol TE/g).

### 4.8. Anti-inflammatory Assay

In a typical PDE inhibitory test [36], the 4-NPP substrate (10 μL, 0.33 mM in PBS), oil sample (5 mg/mL in acetone), and 0.35 μU PDE (in PBS buffer) were mixed in the wells of a 96-well plate. After incubation at 37 °C for 45 min, the optical density (OD) value of reactant was measured at 405 nm on a Thermo Multiskan MK3 reader, and the inhibition efficiency was calculated from the OD value using the following equation:

(2) $$IE\%=(\frac{OD_{\mathrm{bl}}-OD_{s}}{OD_{s}})\times 100\%$$

where the subscripts s and bl indicate sample (UT and FT oil) and reagent blank, respectively. The strong inhibitor EDTA and weak inhibitor L-cysteine were used as positive comparison.

The LOX inhibitory test was modified from a previous study [37]. In a typical measurement,40 μL LOX-5 solution (50 times dilution in water) was incubated at 30 °C for 45 min with a 20 μL oil sample (200 μg/mL in acetonitrile). Then 50 μL linoleic acid was added, and after incubation for 3 min at 30 °C, 500 μL ethanol and 500 μL water were added to terminate the reaction. Then the reactant was measured at 234 nm on a Thermo Multiskan MK3 reader, and the OD value was used to calculate the inhibition efficiency (IE%) by Equation (2). Here, the subscripts s and bl indicate sample and reagent blank, respectively. Nordihydroguaiatic acid (NGDA, IE% = 100%, IC_50_ = 2.95 ± 0.21 μg/mL) [28] was used as a positive control to calculate the IE% of oil samples. The formation of hydroperoxide conjugated dienes gave strong absorption at 234 nm (ε > 25,000 M^−1^.cm^−1^). The interference of sciadonic acid (polyene structure, ε_220–260 nm_ < 1000 M^−1^ cm^−1^) on the test results can be omitted.

In an anti-edema test, 75 mice (37 males and 38 females) were divided randomly into five groups (n = 15: control, FT oil, UT oil, reference, and positive groups). UT oil with the same tocopherols content as FT oil was the reference, and aspirin (total dosage 0.2 mg/cm^2^ on ear) was the positive group. Inflammation was induced by dosing with 0.05 mL of xylene on both sides of the ears (right ear as control). After 30 min of xylene contamination, oil samples (0.05 mL) and aspirin solution were applied to the left and right ears at 1 h intervals over 12 h. The mice were killed, and an 8 mm diameter patch was punched into the left (weight, *W*_l_) and right (weight, *W*_r_) ears; the weight margin of the 2 patches was calculated as the swelling inhibition degree [38] (IE_sw_%):

(3) $$IE_{\mathrm{sw}}\%=(\frac{W_{l}-W_{r}}{W_{r}})\times 100\%$$

### 4.9. Statistical Analysis 

All oil samples were tested in triplicate and in a random order. To indicate the difference of data within a group, multiple comparison was performed by one-way ANOVA and, successively, Tukey’s test using SPSS Statistics 15.0 (SPSS Inc., Chicago, IL, USA) at a P-value less than or equal to 0.05. Student’s *t* test was used to compare the statistical significance between the original UT oil and fractionated FT oil.

## Figures and Tables

**Table 1:** Properties and fatty acid (content higher than 1%) composition of different Torreya kernel oils.

	*T. fragesii* Oil (UT Oil)	Fractionated *T. fragesii* Oil (FT Oil)	*T. grandis* Oil
Color (Lovibond 1.0 inch)	Y30 + R1	Y30 + R0.5	-
Viscority 20 °C, mPa.s	55–90	60–90	-
Density, 20 °C, g/cm^3^	0.920–0.930	0.910–0.930	-
Acid value (AV), mg KOH/g	0.35 ± 0.01 ^a^	0.33 ± 0.01 ^a^	0.2–3
Peroxide value (PV), meq/g	0.56 ± 0.05 ^a^	0.49 ± 0.04 ^a^	0.1–1
Iodine value (IV), g I_2_/100g	145 ± 1.8 ^a^	161 ± 2.0 ^b^	130–150
Saponification value, mg KOH/g	186 ± 2.3 ^a^	188 ± 2.6 ^a^	180–200
Unsaponifiable matter, %	0.82 ± 0.05 ^a^	0.76 ± 0.05 ^a^	-
Wax, %	2.12 ± 0.25 ^a^	0.05 ± 0.00 ^b^	-
C16:0, % by GC, same as below	9.68 ± 0.35 ^a^	2.62 ± 0.25 ^b^	7–10
C18:0	5.32 ± 0.23 ^a^	3.21 ± 0.21 ^b^	2–4
C18:1	29.36 ± 0.7 ^a^	20.62 ± 0.8 ^b^	17–33
C18:2	39.95 ± 0.3 ^a^	50.68 ± 0.2 ^b^	40–47
C18:3 9c 12c 15c	1.65 ± 0.10 ^a^	2.58 ± 0.31 ^b^	0.4–1
C20:2 11c 14c	1.98 ± 0.14 ^a^	2.35 ± 0.16 ^a^	2–4
C20:3 5c 1c 14c	11.23 ± 0.43 ^a^	25.23 ± 0.45 ^b^	9–18
SFA ^**^	15.00 ± 0.51 ^a^	5.83 ± 0.48 ^b^	10–12
MUFA ^**^	29.36 ± 1.0 ^a^	23.62 ± 1.2 ^b^	19–35
PUFA ^**^	56.64 ± 1.5 ^a^	77.76 ± 1.4 ^b^	53–67
UFA ^**^	85.00 ± 1.9 ^a^	94.17 ± 1.3 ^b^	87–89
Total tocopherols, mg/Kg	1830 ± 25 ^a^	2020 ± 32 ^b^	1400–2300
Total polyphenols, mg GAE^**^/kg	5.6 ± 0.4 ^a^	2.3 ±0.3 ^b^	3.7–31.1
Squalene, mg/kg	15.6 ± 0.05 ^a^	16.2 ± 0.06 ^a^	16–35
Total phytosterols, mg/kg	2020 ± 80 ^a^	1980 ± 75 ^a^	1200–2200

* Values in the same row with different superscript letters are significant difference at *p* < 0.05. ** SFA:saturated fatty acid; MUFA: monounsaturated fatty acid; PUFA:polyunsaturated fatty acid; UFA:unsaturated fatty acid; GAE:gallic acid equivalent.

**Table 2:** The properties of kernel oil after different dewaxing process (WP) and fractionation process (FP).

Dewaxing/Fractionation Condition	Yield of Liquid Fraction %	Sciadonic Acid %	Melting Range (Liquid Fraction)	Solid Fat Content (25 °C, Liquid Fraction)	Wax%
UT oil, original	-	11.2 ± 0.8 ^a^	13.5 ^d^–16.8 ^d^	10.9 ± 1.3 ^c^	2.12 ± 0.25 ^e^
**WP** 2 °C/h (40–25 °C), filtration	96.8 ± 1.6 ^d^	11.5 ± 1.3 ^a^	12.3 ^d^–15.6 ^d^	10.5 ± 1.6 ^c^	0.45 ± 0.09 ^d^
**FP1** 0.5 °C/h, (30–5 °C), 2 rpm	76.6 ± 1.3 ^a^	19.7 ± 0.8 ^d^	4.3 ^b^–7.8 ^b^	2.1 ± 0.8 ^a^	0.06 ± 0.00 ^a^
**FP2** 1 °C/h, (30–5 °C), 2 rpm	80.7 ± 1.8 ^b^	15.3 ± 0.9 ^c^	4.6 ^b^–8.3 ^b^	2.6 ± 0.6 ^b^	0.12 ± 0.02 ^b^
**FP 3** (FT oil) 1 °C/h, (30–15 °C), 0.5 °C/h, (15–5 °C), 2 rpm	75.2 ± 1.7 ^a^	25.2 ± 0.9 ^d^	3.2 ^a^–5.6 ^a^	1.9 ± 0.8 ^a^	0.05 ± 0.00 ^a^
**FP4** 1 °C /h, (30–15 °C), 0.5 °C/h, (15–5 °C), 4 rpm	88.6 ± 2.3 ^c^	13.9 ± 1.5 ^b^	5.5 ^c^–9.2 ^c^	2.5 ± 0.3 ^b^	0.18 ± 0.02 ^c^

* Values in the same column with different superscript letters are significant difference at *p* < 0.05.

**Table 3:** Scavenging ability and LOX-5 inhibition efficiency of different *Torreya* kernel oils.

	UT Oil	FT Oil	EGCG^**^	*T. grandis* Oil	BHT	NDGA^**^
ORAC μmol TE/100 g	445 ± 12 ^a^	458 ± 10 ^a^	8000 ± 124	260–435	-	-
DPPH μmol TE/100 g	485 ± 15 ^a^	502 ± 18 ^a^	-	422–509	-	-
DPPH IC_50_ μg/ml	6.12 ± 0.05 ^a^	5.90 ± 0.12 ^a^	90	-	1.75 ± 0.03	-
LOX-5 IE% (Concentration of oil)	32.1 ± 2.3 ^a^ (66.7 μg/mL)	65.2 ± 3.1 ^b^ (66.7μg/mL)	-	-	-	100 ± 0 (10 μg/mL)
LOX-5 IC_50_ μg/ml	-	47.8 ± 1.3	-	-	-	2.95 ± 0.21

* Values in the same row with different superscript letters are significant difference at *p* < 0.05. ** EGCG: epigallocatechin gallate; BHT:2,6-di-*tert*-butyl-4-methyl phenol; NDGA: nordihydro-guaiaretic acid.

## Supplementary Materials

The following are available online, Figure S1: DSC cooling profile (−3 °C/min) of UT oil (exothermal peaks are shown upwards).
