# Supplementary material for: Fractionated Antioxidant and Anti-inflammatory Kernel Oil from Torreya fargesii
Source: Molecules. 2019 Sep 19;24(18):3402. doi: 10.3390/molecules24183402 (PMC6767029; doi:10.3390/molecules24183402)
Supplement: Supplementary file 1 [file molecules-24-03402-s001.pdf]

*Supplementary Material*

# Fractionated Antioxidant and Anti-inflammatory Kernel Oil from *Torreya fargesii*

**Xianrong Zhou *, Jin Shang, Mingyi Qin, Jianhua Wang, Bo Jiang, Hui Yang and Yan Zhang**

School of Advanced Agriculture and Bioengineering, Yangtze Normal University, Fuling 408100, P.R. China
*Correspondence: zxrfssy@outlook.com

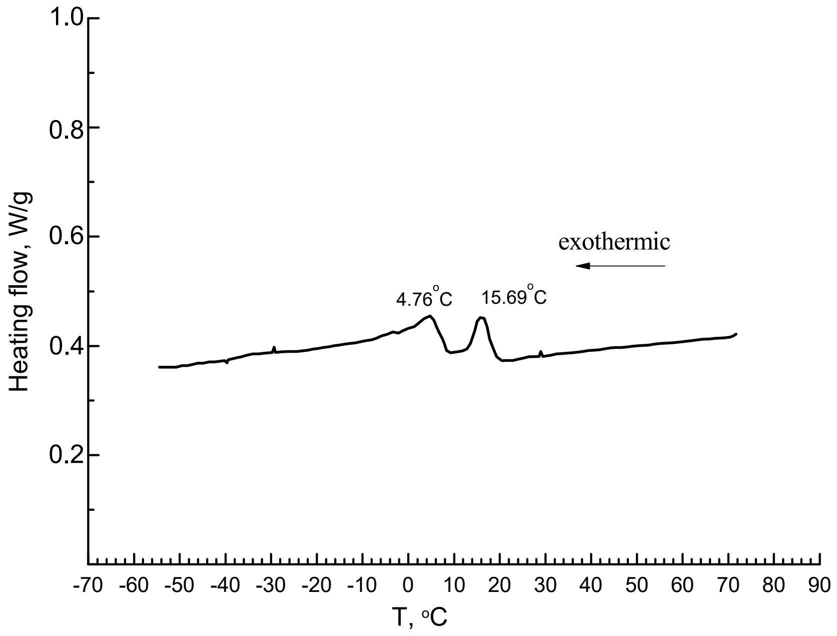


**Supplemental Figure S1.** DSC cooling profile (-3 °C/min) of UT oil (exothermal peaks are shown upwards).
